# Study on sustainable developments in Guangdong Province from 2013 to 2018 based on an improved ecological footprint model

**DOI:** 10.1038/s41598-022-06152-4

**Published:** 2022-02-10

**Authors:** Ye Liu, Xi Zhou, Qiuyun Zhang, Lixuan Zeng, Yuan Kang, Jiwen Luo

**Affiliations:** School of Environment, South China Normal University, Guangzhou, 510006 Guangdong China

**Keywords:** Sustainability, Environmental impact

## Abstract

Aiming at the ecological footprint model, the traditional trade adjustment method only considered the international trade process at the urban scale, ignoring the trade footprint generated by domestic trade and indirect trade in various products. This paper adopts the urban-scale ecological footprint model based on the macro-trade adjustment method to calculate the trade adjustment coefficient of biological products and the energy trade adjustment coefficient respectively to correct the trade footprint. The results showed that the per capita ecological deficit showed a straight upward trend, from 0.07351 hm^2^ in 2013 to 0.15472 hm^2^ in 2018. From 2013 to 2018, the per capita ecological footprint of Guangdong Province was greater than the per capita ecological carrying capacity, and the ecological economic system of Guangdong Province was in an unsustainable state. According to the trade ecological footprint, Guangdong Province was a completely foreign resource and service exporting city, which was consistent with Guangdong Province’s own economic development direction; the analysis results of the ecological product trade footprint were more consistent with the current city positioning of biological resource products of each city, and the energy indirect trade footprint. The improved ecological footprint model could more accurately assess the true status of ecological vitality above the urban scale.

## Introduction

Ecological footprint, also known as “ecological occupation”, refers to the sum of all the resources consumed by a certain region (country or region) and the area of bioproductive land (covering land and water) needed to absorb the waste generated by the region. It was first proposed by Canadian economist William E.Rees in 1992^1^ and improved by his student Wackernagel in 1997^2^. Xu Zhongmin et.al. introduced the ecological footprint model and calculation method into China for the first time in 2000, and carried out empirical calculation and analysis on the ecological footprint of Gansu Province in 1998^3^. Due to its advantages of relative convenience in obtaining data, simple calculation method and intuitive results, it has been widely quoted by scholars at home and abroad. Up to now, many scholars has made improvements on the basis of the original basic models, such as emergy-based ecological footprint model^4–6^, input–output footprint model^7,8^, time series ecological footprint model^9–11^, macro trade adjustment model^12,13^ and three-dimensional ecological footprint model^14–16^. The research scale was gradually narrowed from global^17^, national^18^, regional^19^, industry^20^ and school^21^. With the increasingly obvious trend of economic globalization and trade liberalization, the ecological burden transfer caused by trade has gradually attracted people's attention^22^. After continuous exploration by scholars, ecological footprint method has gradually been used as a method to measure and reflect the changes of ecological environment between international trade. It converted import and export commodities into the biological production-type land area which was required for production and consumption of commodities, and pursued the surplus of ecological footprint in trade^23^. Andersson and Lindroth believed that some relatively developed countries kept importing commodities from developing countries to maintain their local biocapacities by taking advantage of their economic advantages and transferring the environmental burden to other countries, which further worsened the ecological environment of developing countries. To some extent, this was a serious ecological inequitable exchange^24^. Zhang Xueqin and Chen Chengzhong used the input–output method and emergy method respectively to calculate the ecological footprint of China's import and export trade from 1995 to 2005, and dynamically analyzed the impact of international trade on China's ecological environment^25^. Lu Changgeng and Zhao Yichen used emergy theory to measure the ecological footprint in international trade and analyzed the influencing factors of import and export trade^26^. Zhang Chuanguo and Qu Xuqin used the ecological footprint accounting method to study the sustainable development of trade between China and Russia, and conducted an empirical study on the structure and dynamic changes of the trade ecological footprint of the two countries in this period^27^. Dai Haitian and Chen Fang analyzed the ecological footprint of foreign trade in Anhui province and its influencing factors^28^. However, traditional net trade often used the international ecological footprint model (production = import–export) to achieve trade adjustment. It only considered the impact of trade between different countries, while ignored the trade process of trade and processed products between different cities, and did not reflect the impact of urban consumption on the ecological environment in the process of urban development. This paper adopted the urban-scale ecological footprint model based on macro-trade correction method to adjust the ecological footprint of Guangdong Province, and analyzed the overall impact of trade and processing trade processes between Guangdong Province and cities on the ecological footprint of Guangdong Province. The calculation results of Guangdong Province's ecological footprint were closer to reality, so as to provide theoretical basis for Guangdong Province to achieve sustainable development.

**Figure 1:**
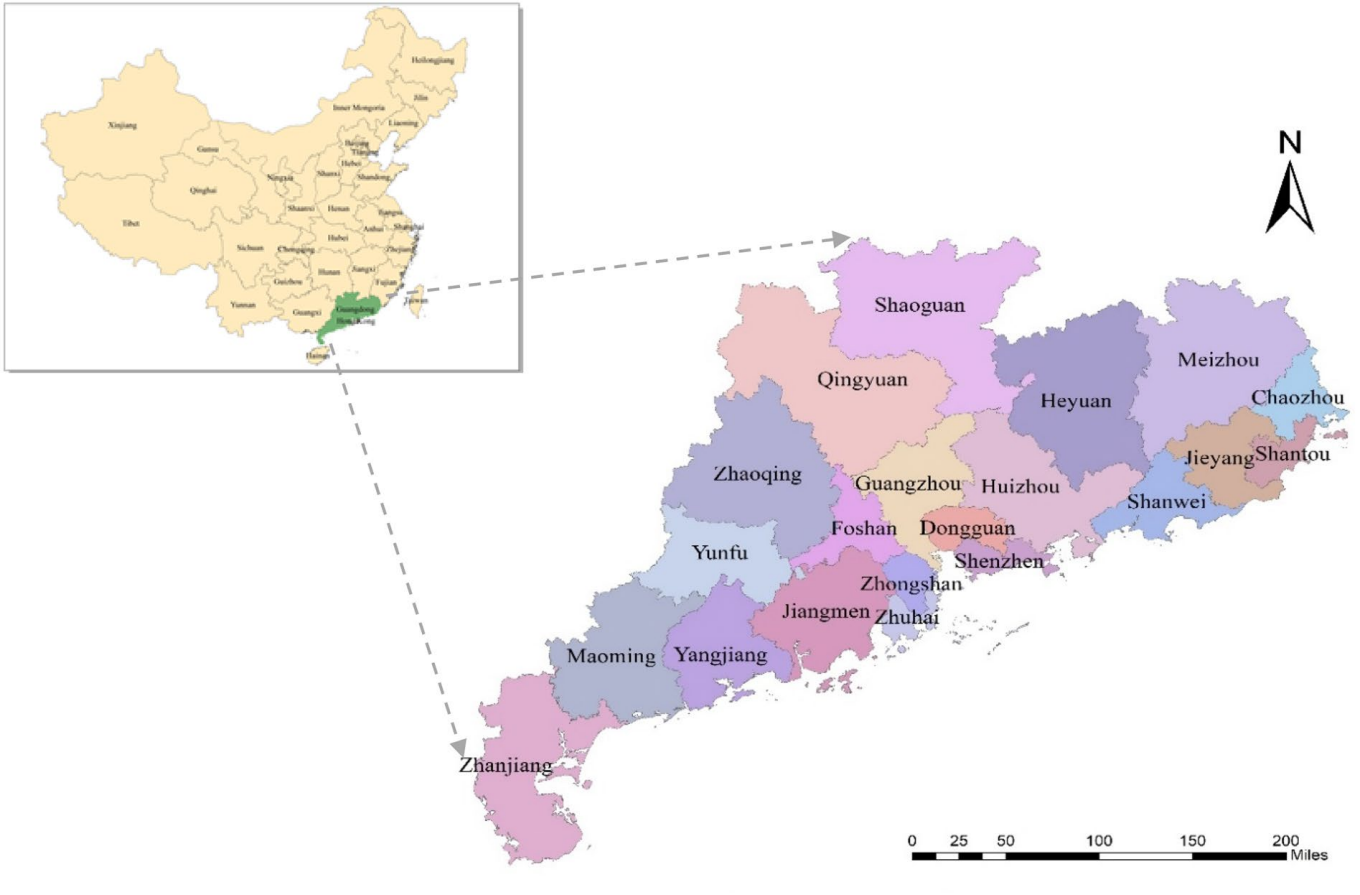
Geographical location of Guangdong Province and its 21 cities. Figure was made in ArcGIS 10.2 (https://support.ersi.com/en/dowload/2093). The dada source of administrative and city names was derived from the online open-source data.

Guangdong Province is located in the southernmost part of China’s mainland, whose land range is located at 20°09'–25°31' north latitude and 109°45'–117°20' east longitude. It is in the south of Nan ling mountain, on the shore of the South China Sea, facing Hong Kong and Macao Special Administrative Region across the sea (Fig. 1). It belongs to the East Asian monsoon region, with subtropical and tropical climates from north to south. It is one of the most abundant areas with light, heat and water resources in China. As a pioneer of reform and opening-up, Guangdong's GDP has increased from 18.6 billion yuan in 1978 to 9.73 trillion yuan in 2018, with an average annual growth rate of 12.5%, ranking first in China during 30 consecutive years.

With the introduction of foreign capita and advanced technology, Guangdong's economy had developed rapidly; people from outside Guangdong Province continue to flow into Guangdong, and the urbanization rate continues to increase. 2013–2018 was a period of acceleration of urbanization development. With the economic growth of Guangdong Province and the improvement of people's living standards, the demand for natural resources and the pressure on the local natural environment was increasing gradually, which has restricted the sustainable development of Guangdong Province. How to improve local resource utilization efficiency and sustainable development capacity has become a top priority.

## Data sources

The impact of human beings on the local ecosystem was mainly reflected in the production and living consumption, including the consumption of biological resources and energy. Biological resources consumption included agricultural products, livestock products and aquatic products. Energy consumption included coal, coke, crude oil, fuel oil, gasoline, kerosene, diesel and electricity. Agricultural products included grain, vegetables and fruits. As the marine and freshwater fishing area was difficult to obtain, there was no statistical data on the catch of aquatic products in the Guangdong Provincial Yearbook, and the consumption of aquaculture was a major part of local consumption. This article only considered the aquaculture in the water area. Livestock products included pork, beef, lamb, poultry, eggs, milk, etc. The consumption of livestock products was based on the proportion of the substances to determine the materials consumed by livestock products, and then the ecological footprint of livestock products was calculated according to the attribution of the ecological production land of these substances^29^. The data of biological resources and energy consumption came from Guangdong Statistical Yearbook^30^, China Statistical Yearbook^31^, China Electricity Yearbook^32^ and China Fishery Statistical Yearbook^33^ from 2014 to 2019. Land use was divided into six categories: cultivated land, forest land, grassland, construction land, water area and fossil energy land^17^. The fossil energy land was forest land and grassland used to absorb greenhouse gases emitted by burning fossil energy^34^. The national average yield was derived from the FAO database^35^.

## Methods

### The ecological footprint

1 $$ Ef = N \times ef = N \times r_{j} \times y_{j} \times \mathop \sum \limits_{i}^{n} aa_{i} = N \times r_{j} \times y_{j} \times \mathop \sum \limits_{i}^{n} \left( {\frac{{c_{i} }}{{EP_{i} }}} \right) $$

In Eq. (1), *Ef* is the ecological footprint before trade adjustment of the province (hm^2^)^29^, *N* is the total population of the province, *ef* represents the province's per capita ecological footprint (hm^2^), *i* is the consumption type in accounting, *aa*_*i*_ is the actual ecologically production land area per capita occupied by the consumption in item *i* (hm^2^), *c*_*i*_ is the per capita annual consumption in item *i*, *EP*_*i*_ is the annual national average output of the consumer goods in item *i* (kg/hm^2^), *y*_*j*_ is the yield adjustment factor, *r*_*j*_ is the land use balance factor, *j* is the type of ecological productive land, among them, j = 1, 2, 3, 4, 5, 6 represent fossil energy land, cultivated land, grassland, forest land, water area and completed land respectively.

### Ecological capacity

Measuring ecological capacity by ecological footprint refers to the premise of not compromising the productivity and functional integrity of ecological systems. The total area of ecologically productive land that a region can own. That is, the ecological carrying capacity of the region. This paper adopts the calculation formula proposed by Xie Hongyu and other scholars to calculate ecological capacity based on the actual ecological product output and the ecological service capacity provided by the land within one year^17^.

2 $$ AC = \mathop \sum \limits_{i}^{n} \frac{{P_{i} }}{{\overline{{EP_{i} }} }} \times r_{j} $$

In Eq. (2), *AC* is the ecological capacity (hm^2^), *P*_*i*_ is the resource production quantity of the *i* ecological product in the *j* ecological productive land (kg), $\overline{{EP_{{\text{i}}} }}$ is the national average single yield (kg/hm^2^) of the *i* kind of ecological product in the *j* category of ecological productive land, *r*_*j*_ is the land use balance factor, *j* is the type of ecological productive land (*j* = 1, 2, 3, 4, 5, 6).

### Yield adjustment and equalization

Because the productivity of similar ecological productive land varies from country to country and region, the actual area of similar ecological productive land in various countries and regions cannot be directly compared and needs to be adjusted by multiplying the area of its ecological productive land by the yield factor. Because the productivity of each type of land is different, the area of each type of land should be multiplied by its own equivalent factors, and then the equivalent area of each type should be added to obtain the value of the regional ecological footprint and capacity^36^.

3 $$ y_{j} = \frac{{P_{j} }}{{EP_{j} }} $$

In Eq. (3), *P*_*j*_ is the production of class j ecologically productive land in the region, *EP*_*j*_ is the national production of ecologically productive land in category *j*. In order to accurately reflect the different production capacities of different land use types in Guangdong Province, the equilibrium factor of Guangdong Province calculated by Liu Moucheng based on the primary productivity^37^ is selected (Cultivated land is 1.36, Forest land is 0.68, Grass land is 0.57, Water area is 0.45, and Construction land is 1.36).

### Trade adjustment

This study refers to the macro-trade adjustment method proposed by Bai Yu et. al. to adjust the ecological footprint of Guangdong Province^13^. The calculation method is mentioned in Eqs. (4–6)

4 $$ EF = c_{b} \times Ef_{b} + c_{e} \times Ef_{e} $$

In Eq. (4), *EF* is the total ecological footprint consumption, *c*_*b*_ is the trade adjustment coefficient of the biological resources account, *Ef*_*b*_ is the ecological footprint of the biological resources account, *c*_*e*_ is the trade adjustment coefficient of energy account, *Ef*_*e*_ is the ecological footprint of the energy account.

5 $$ c_{b} = \frac{EC \times H}{{G_{p} + G_{F} }} $$

6 $$ c_{e} = \left( {\frac{{E_{F} }}{E} \times \frac{EC \times H}{{G_{F} }} + \frac{{E - E_{F} }}{E} \times \frac{C - EC \times H}{{G - G_{F} }}} \right) \times \frac{G}{G - A - W} $$

In Eqs. (5) and (6), *EC* is Engel's coefficient (the proportion of total food expenditure to total personal consumption expenditure), *H* is household consumption expenditure, *G*_*p*_ is GDP of primary industry, *G*_*F*_ is GDP of food processing industry, including agricultural and sideline product processing industry, food manufacturing industry and beverage manufacturing industry, *E* is the total energy consumption, *EF* is the energy consumption of the food industry, *C* is total final consumption, including residents' consumption and government consumption, *G* is gross national product, *A* is the total investment in fixed assets, *W* is the total wage of workers.

### Ecological deficit and ecological surplus

Comparing the ecological footprint occupied by the resources, energy consumption, and waste discharge of a region or country with the ecological capacity it owns, there will be an ecological deficit (the ecological footprint is greater than the ecological capacity, which means that the human load in the region exceeds its ecological capacity and shows an unsustainable state) and ecological surplus (the ecological footprint is less than the ecological capacity, which means that the ecological capacity of the area is sufficient to support its human load and is in a sustainable state)^2^.

7 $$ ED/ES = EF - AC $$

In Eq. (7), *ED* is ecological deficit, *ES* is ecological surplus.

### Ecological footprint of ¥10,000 GDP

To better reflect the local natural resources utilization efficiency in Guangdong Province, this study combines the ecological footprint of ¥10,000 GDP to conduct a comprehensive analysis of the local resident’s consumption. The lower the ecological footprint of ¥10,000 GDP, the higher the resource utilization efficiency, and vice versa.

8 $$ WEF = \frac{Ecological\, footprint \,per \,capita}{{GDP\, per \,capita}} \times 10,000 $$

In Eq. (8), *WEF* is ecological footprint of ¥10,000 GDP.

## Result

### Biological resource account calculation

**Figure 2:**
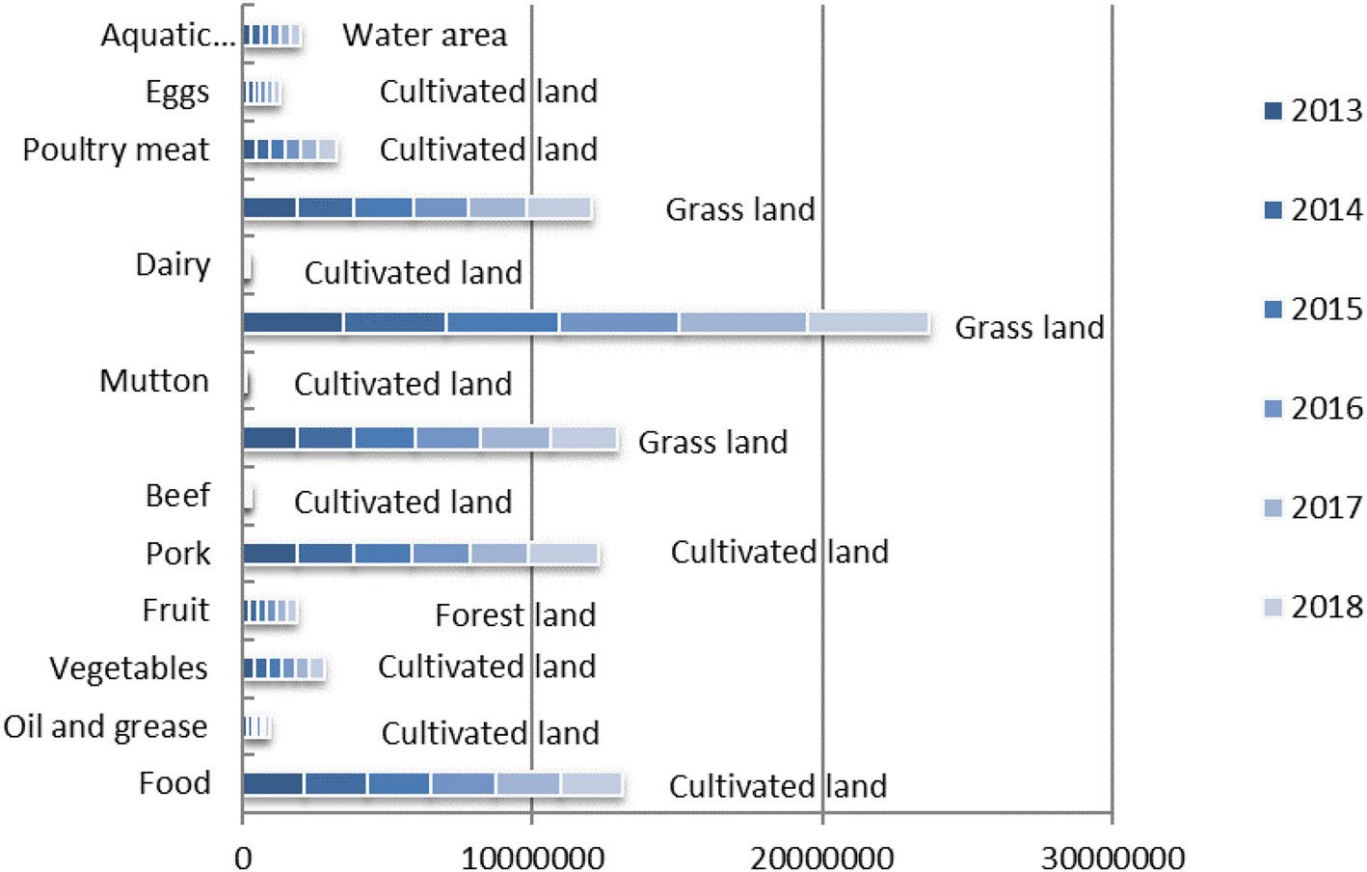
Ecological footprint of biological resource accounts in Guangdong Province from 2013 to 2018 (hm^2^).

The consumption of biological resources included the consumption of agricultural products, livestock products and aquatic products. According to Xie Hongyu’s consumption of raw materials of livestock products per kg^29^, the ecological footprint of livestock products per kg could be calculated by combining the average output data of raw materials in China, and the ecological footprint of biological resources consumption in Guangdong Province from 2013 to 2018 could be calculated by combining the consumption data of local residents in Guangdong Province, and the result was shown in Fig. 2.

### Calculation of energy account

**Figure 3:**
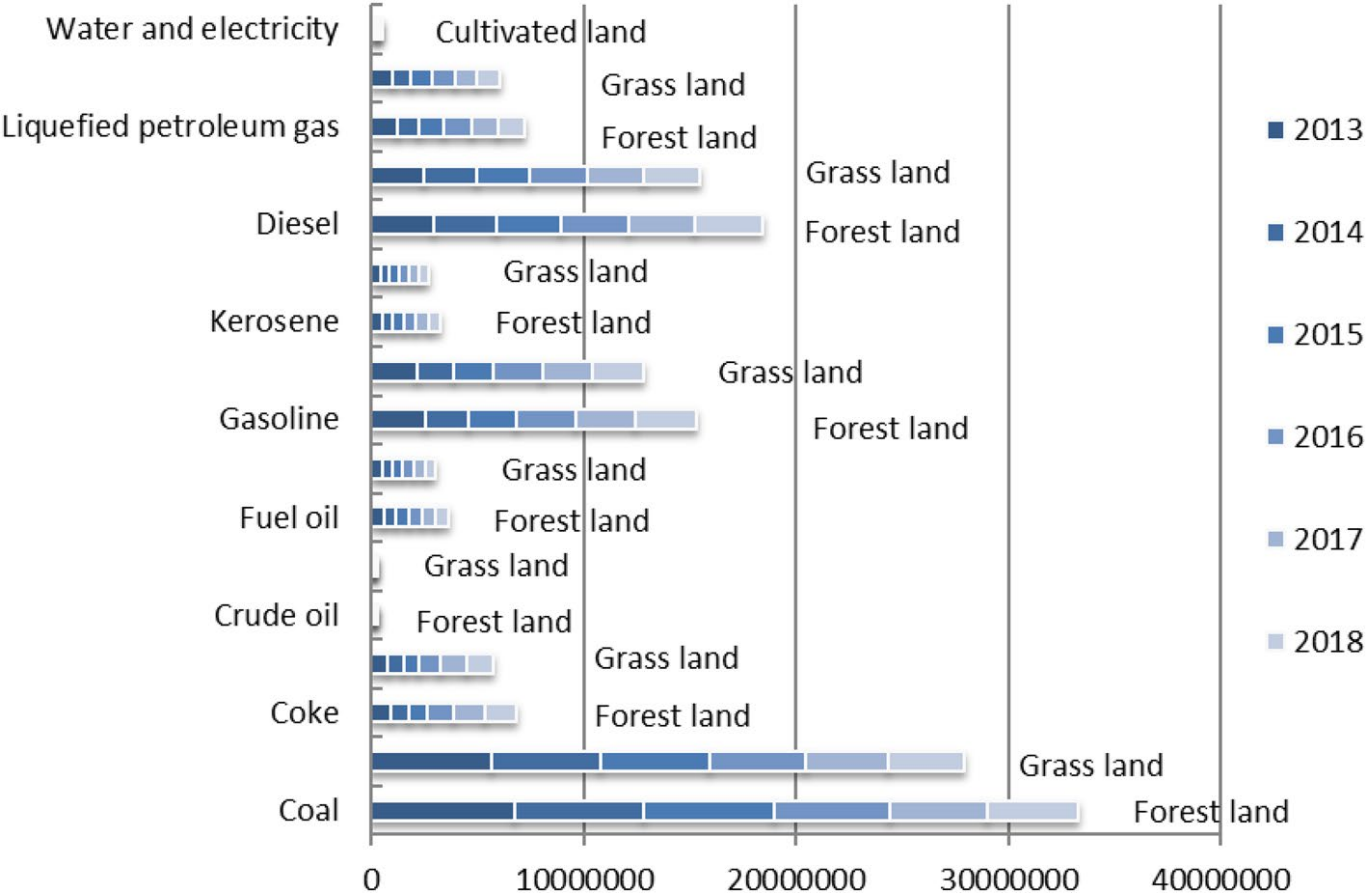
Ecological footprint of energy accounts in Guangdong Province from 2013 to 2018 (hm^2^).

1. Fossil energy
  According to the ecosystem cycle theory, forest land accounted for about 77% of global terrestrial surface vegetation reserves, while grassland carbon reserves accounted for about 16% of global terrestrial surface vegetation reserves. Although the two were far apart, they should not be ignored. With reference to the research of Xie Hongyu and other scholars defined fossil energy land as forests and grasslands that absorb greenhouse gases emitted by the burning of fossil energy, the fossil energy footprint of Guangdong Province was calculated based on its unit fossil energy footprint and actual consumption^34^, which was given in Fig. 3.
2. Electric power
  At present, there were two main power generation modes in China, namely thermal power generation and hydroelectric power generation. Given that the main fuel of thermal power in China was fossil energy, there was a certain overlap with fossil energy consumption. To avoid double counting of fossil energy, the thermal power consumption was removed from the electricity consumption in Guangdong Province, but only hydroelectric consumption was considered. However, the statistical yearbook of Guangdong Province had total electricity consumption, and did not show hydropower consumption data. The annual hydropower consumption of Guangdong Province was obtained through the proportion of hydropower generation in the total electricity generation. Combined with the research of Xie Hongyu and other scholars, the ecological footprint of 1 kWh hydropower in China was 2.1448 × 10^–6^ hm^2^ arable land^21^. The electric power ecological footprint of Guangdong Province from 2013 to 2018 was calculated and shown in Fig. 3.

### Trade adjustment

This study refers to the macro-trade adjustment method proposed by Bai Yu and other scholars^12^. The trade adjustment coefficient of the biological resources account and the energy account of Guangdong Province were calculated. The annual trade adjustment coefficient of the biological resources accounted and the energy account of Guangdong Province were calculated by collecting the statistical data of Guangdong Province from 2013 to 2018. The results were given in Table 1.

**Table 1:** Trade adjustment coefficients of Guangdong Province from 2013 to 2018.

Project	Year				
	2013	2014	2015	2017	2018
Biological resources account trade adjustment factor	0.97	1.01	1.04	1.18	1.18
The trade adjustment factor for the energy account	0.86	0.91	1.02	1.01	1.09

### Analysis of ecological footprint composition

**Figure 4:**
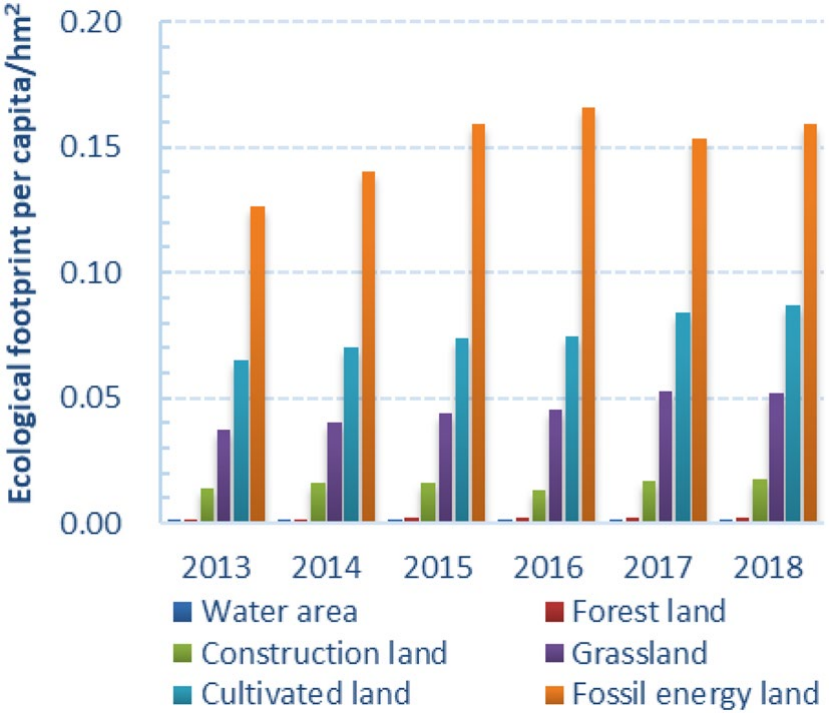
Trends of six types of ecologically productive land in Guangdong Province from 2013 to 2018.

**Figure 5:**
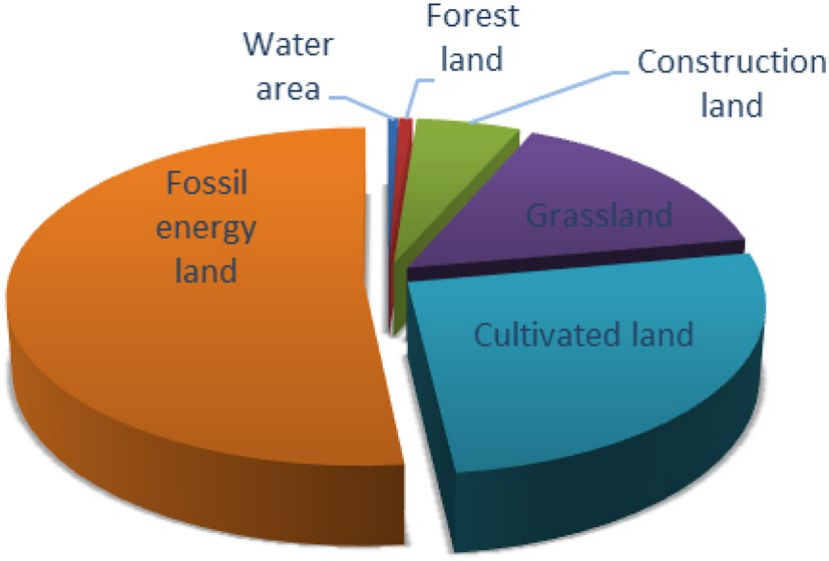
Share of different types of land in total ecological footprint.

**Figure 6:**
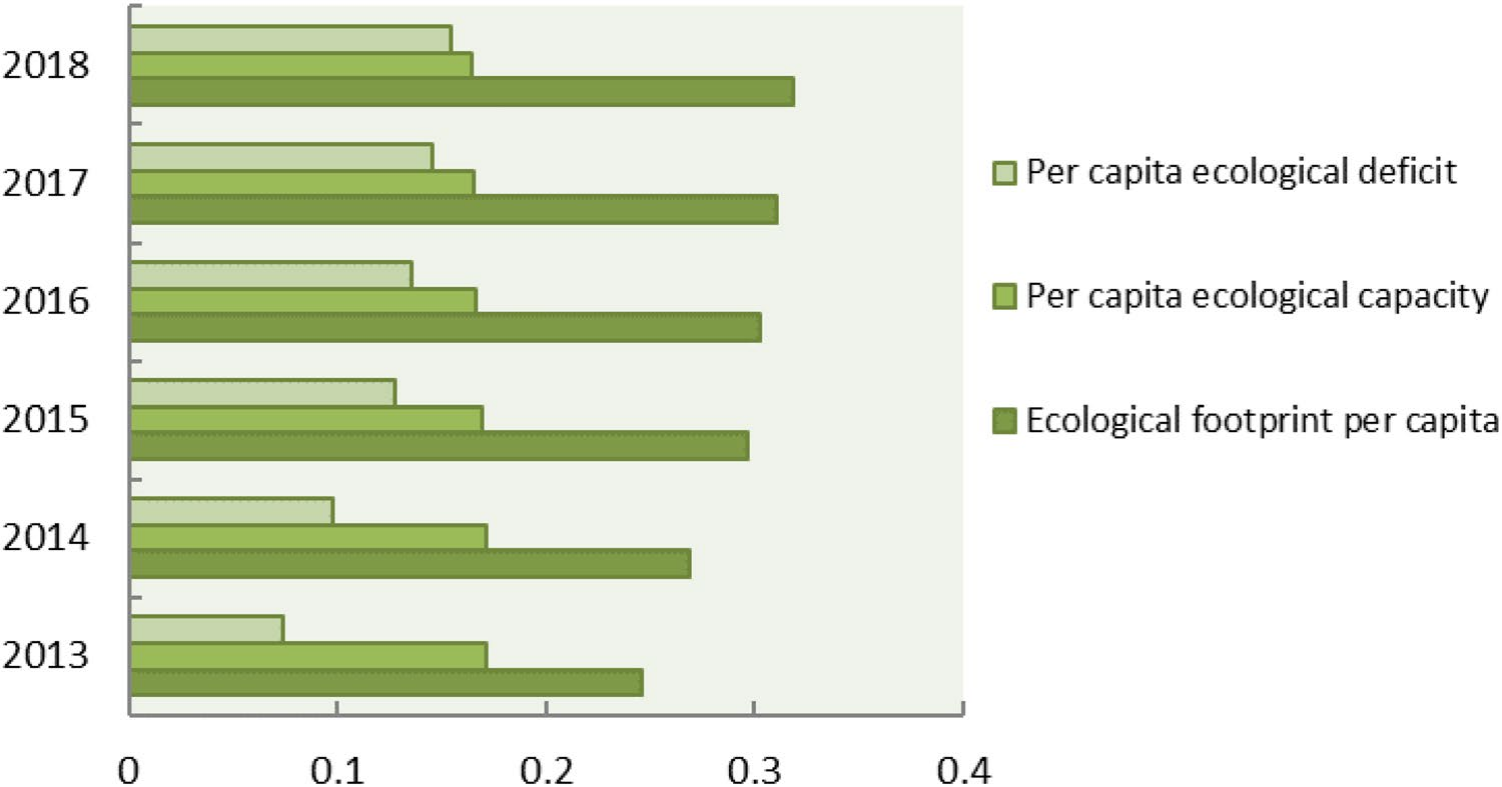
Dynamic changes of per capita ecological footprint, ecological carrying capacity and ecological deficit in Guangdong Province from 2013 to 2018.

In Figs. 4 and 6, the per capita ecological footprint of Guangdong Province has shown an obvious growth trend, increasing from 0.24577 hm^2^ in 2013 to 0.31911 hm^2^ in 2018. The per capita ecological footprint of different types of ecological productive land generally has shown a growth trend in the past six years. The proportions of the six kinds of ecological productive land type per capita ecological footprint were fossil energy land > cultivated land > grassland > construction land > forest land > water area (Fig. 5). Guangdong’s high demand for energy and agricultural products was the main reason for the high ecological footprint. Among them, the fossil energy consumption caused by urbanization, industrial transformation, the improvement of people's living quality, as well as the popularity of cars was the biggest reason. At the same time, the consumption of cultivated land and grassland also accounted for a large proportion. The reason was that with the improvement of living standards, people's demand for agricultural products and meat products was also increasing. As the population increases, the land for construction also increased. The forest land and water area account the smallest in the whole ecological footprint, and the influence on the whole ecological footprint was not significant with the small increase. It could be concluded that fossil energy land, cultivated land, grassland and construction land were the main reasons for the change in the per capita ecological footprint, as well as the important reasons for the local ecological deficit.

In Fig. 6, the per capita ecological capacity of Guangdong Province has not changed much, but the overall downward trend was obvious, from 0.17186 hm^2^ in 2013 decreased to 0.16439 hm^2^ in 2018. The per capita ecological deficit showed a linear upward trend, from 0.07391 hm^2^ in 2013 to 0.15472 hm^2^ in 2018. From the perspective of ecological deficit of each component, the fossil energy deficit was the largest, with an average of 0.06567 hm^2^, followed by wood land of 0.03874 hm^2^, and finally construction land of 0.01565 hm^2^. Since 2016, the cultivated land has shown an ecological deficit, and it has been increasing year by year. Both forest land and water area showed ecological surplus, which were 0.01512 hm^2^ and 0.00333 hm^2^, respectively. The ecological surplus of forestland showed a "V" shaped change trend and began to increase linearly in 2016, indicating that the policies such as "returning farmland to forest" have achieved certain results. However, with the development of social economy, the increase of population and the continuous improvement of living standard, Guangdong Province was still in the state of ecological deficit, and the overall trend of increasing ecological footprint has not been fundamentally reversed. It can be concluded that the economic and social development of Guangdong Province from 2013 to 2018 was based on the overdraft of natural ecology and was in an ecologically unsustainable state.

### Analysis of ecological footprint of six prefecture-level cities in Guangdong Province

**Figure 7:**
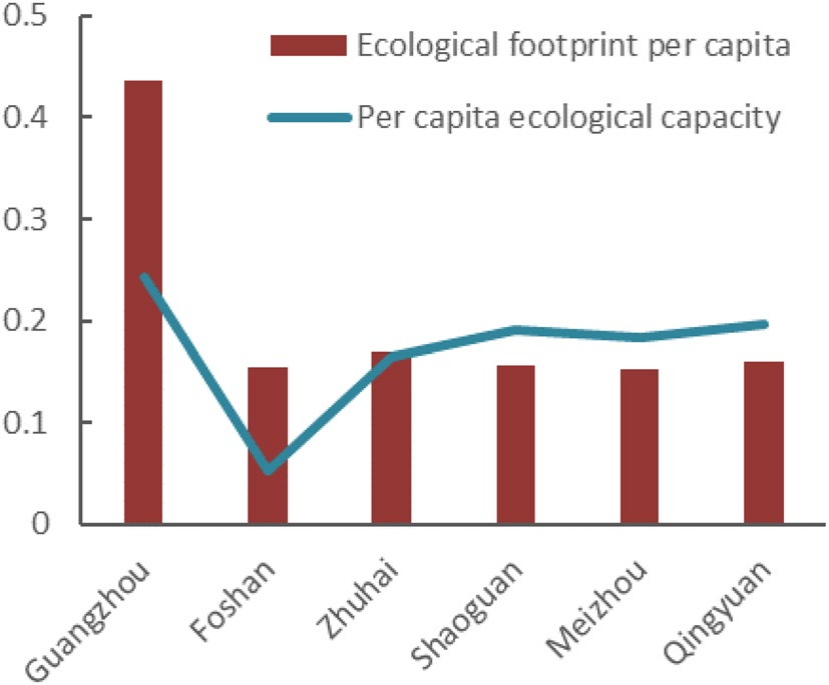
Analysis of ecological footprint of cities in 2013.

**Figure 8:**
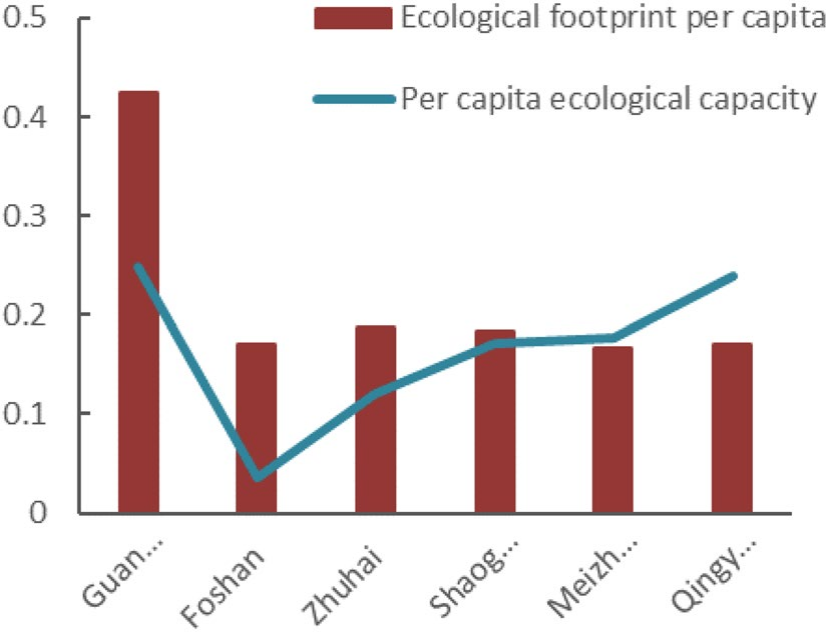
Analysis of ecological footprint of cities in 2018.

This study selects six prefecture-level cities to conduct ecological footprint analysis. Guangzhou, Foshan and Zhuhai were the economically developed regions in Guangdong Province. It could be seen that their per capita ecological footprint was significantly higher than that of the economically backward regions such as Shaoguan, Meizhou and Qingyuan (Figs. 7 and 8). From 2013 to 2018, the regional per capita ecological footprint has shown an increasing trend. From the perspective of per capita ecological capacity, Qingyuan has grown significantly, Guangzhou has not changed much, and other regions have shown a downward trend. Especially in Foshan and Zhuhai, the ecological capacity has dropped significantly and the ecological deficit has soared, suggesting that these regions are in a severely unsustainable state. Besides Qingyuan, the ecological surplus in Shaoguan and Meizhou decreased, and the local consumption also tends to balance. Qingyuan was in the state of ecological surplus, which was related to the adequacy of available local resources and low consumption level belonged to the backward economy. With the rapid growth of Guangdong’s population, the consumption level and the ecological footprint had increased. Through research and analysis, it could be seen that the ecological deficits were concentrated in economically developed areas, while the ecological surpluses were distributed in underdeveloped regions, which might be related to the economic development of Guangdong province. The period from 2013 to 2018 was the accelerated period of urbanization development, resulting in a large income gap between urban and rural residents as well as a different consumption habit, therefore, there were large ecological footprint differences in these regions.

### Analysis of ecological footprint of ¥10,000 GDP

**Figure 9:**
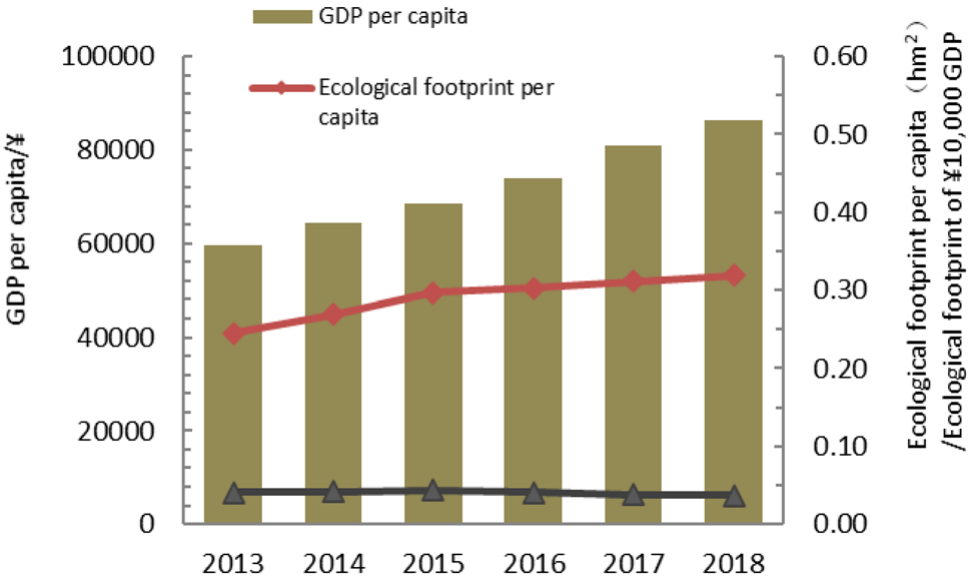
Change of ecological footprint of ¥10,000 GDP in Guangdong Province from 2013 to 2018.

The ecological footprint of ¥10,000 GDP in Guangdong Province fluctuated from 0.04119 hm^2^·mil^-1^ in 2013 to 0.03693 hm^2^·mil^-1^ in 2018, showing a trend of first rising and then falling (Fig. 9). The decrease in the ecological footprint of ¥10,000 GDP reflected the increase in the utilization rate of local resources. Guangdong Province has shown a downward trend since 2016, and the Guangdong resource utilization rate has increased since 2016, which might be related to the policy of "returning farmland to forests" and other policies. The fossil energy has been in a state of ecological deficit for a long time, and the per capita ecological footprint has been increasing year by year. In the future development, we must focus on energy conservation and consumption reduction, reduce the use of fossil energy, optimize and upgrade the industrial structure, and vigorously develop a circular economy to ensure the sustainable development of Guangdong Province.

### Trade footprint analysis

The ecological footprint model after amendment of the macro-trade adjustment method could more comprehensively reflect the proportion of the trade ecological footprint in the ecological footprint, and could better reveal the impact of the trade process on urban development^13^. In this study, the indirect biological products and energy trade footprints generated by Guangdong’s trade were included in the scope of trade increments, so that the ecological footprint estimation was more consistent with the actual situation and the impact of residents on the ecological environment was more accurate. For the biological resource account, the difference in the calculation of ecological footprint before and after the adjustment was the net export trade footprint of biological products. For the energy account, the difference in the calculation of ecological footprint before and after the adjustment was only the ecological footprint of energy in the process of processed product trade, which was the indirect trade footprint of energy^12^.

**Figure 10:**
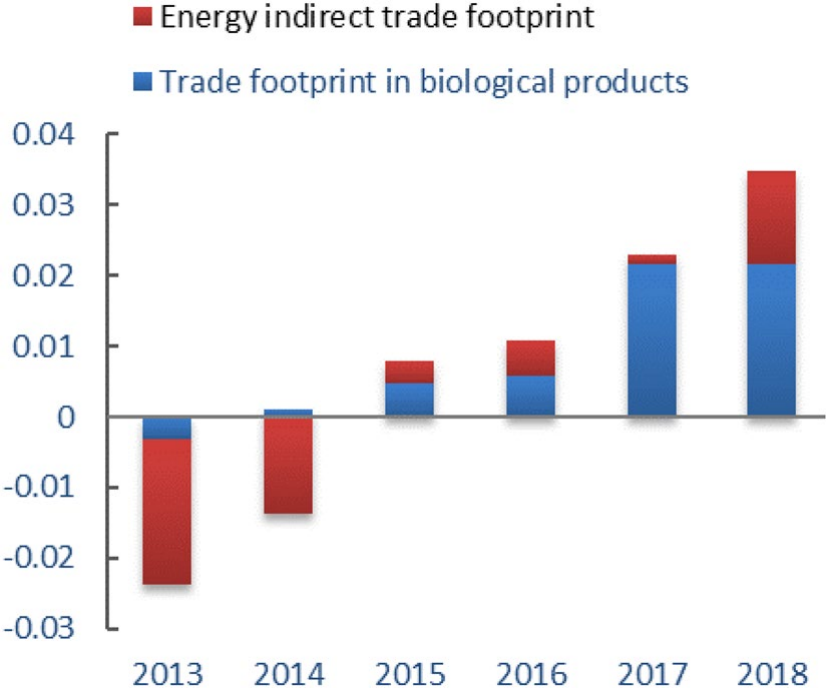
The per capita trade footprint of Guangdong Province from 2013 to 2018.

The per capita trade footprint of Guangdong Province from 2013 to 2018 was shown in Fig. 10. The negative trade footprint of biological products and indirect trade footprint of energy products mean that the import trade footprint was larger than the export trade footprint, and the trade footprint was in deficit, which represented the city's positioning as a recipient of foreign resources and services. On the contrary, it was a surplus of trade footprint, which represented the city's positioning for the export of foreign resources and services^13^. In Fig. 10, the overall ecological product trade footprint of Guangdong Province was surplus. Therefore, Guangdong Province belonged to a city with complete foreign resources and services exports, which was consistent with the economic development direction of Guangdong Province.

**Figure 11:**
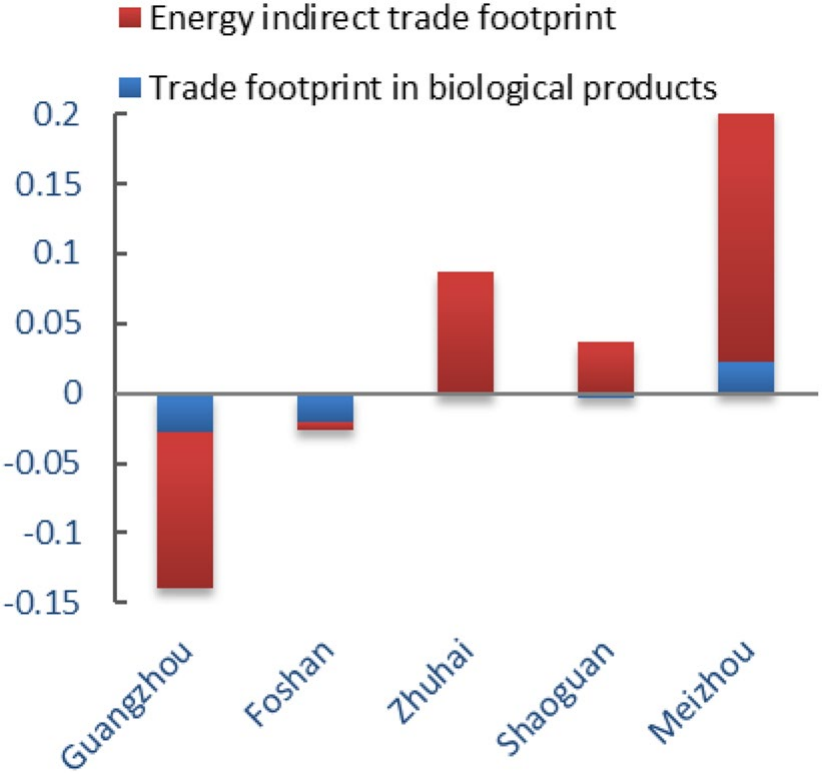
The per capita trade footprint of 5 prefecture-level cities in Guangdong in 2018.

To further analyze the ecological footprint of trade of Guangdong Province, this article selected the Guangzhou, Foshan, Zhuhai, Shaoguan and Meizhou as the analysis objects of the trade footprint of biological products and the indirect footprint of energy products. Only Meizhou belonged to complete external resources and services output type, and the result was shown in Fig. 11. Guangzhou and Foshan were cities that fully receive biological resources services and energy products and services, which was consistent with the positioning form of each city. The indirect trade footprint of energy also truly reflected its current overall economic development mode and regional development characteristics, which was difficult to achieve with traditional ecological footprint estimation methods.

## Discussion

The traditional ecological footprint model essentially only considered the direct international trade of the items counted in the ecological footprint account, ignoring the footprints generated by the mutual trade between cities and the trade of processed products. This paper adopted the ecological footprint adjusted by macro-trade. This model, adding more complex trade processes into the calculation, could more closely reflect the true sustainable state of the study area. However, the ecological footprint after trade adjustments also has shortcomings, especially when calculating the ecological footprint. Due to the difficulty of data acquisition and the inconsistent statistical caliber, the data obtained often have errors and other problems. The data are dynamically analyzed, which are difficult to obtain. In the future, relevant research is still needed to improve the principles and model methods of ecological footprint analysis.

## Conclusion

Based on the macro-trade adjustment of local consumption in Guangdong Province, the study analyzed the per capita trade footprint, per capita ecological footprint, per capita ecological carrying capacity and ecological deficit of Guangdong Province from 2013 to 2018, and analyzed the per capita ecological footprint before and after the trade adjustment, and came to the following conclusions.

1. According to calculations, the per capita ecological footprint of Guangdong Province increased from 0.24577 hm^2^ in 2013 to 0.31911 hm^2^ in 2018, and the per capita carrying capacity decreased from 0.17186 hm^2^ in 2013 to 0.16439 hm^2^ in 2018. The per capita ecological footprint was on the rise, while the per capita carrying capacity was on the decline. The pressure on the local ecosystem increased rapidly, and the ecological deficit was in a state of long-term development. The per capita ecological deficit increased year by year, from 0.07351 hm^2^ in 2013 to 0.15472 hm^2^ in 2018. The ecological footprint of ¥10,000 GDP of Guangdong Province showed an inverted "V" shape during 2013–2018, and the resource utilization rate of Guangdong Province started to rise since 2016, which might be related to the policy of "returning farmland to forests" in Guangdong Province. However, the local fossil energy areas were in a state of ecological deficit for a long time, and the per capita ecological footprint was increasing year by year. In general, from 2013 to 2018, the economic and social development of Guangdong Province was built on the natural ecological overdraft and was in an ecologically unsustainable state.
2. Among them, the proportion of per capita ecological footprint of the six types of ecologically productive land types in the total ecological footprint was in order of fossil energy land, cultivated land, grassland, construction land, forest land, and water area. The ecological deficit of fossil energy land, cultivated land and grassland gradually increased. Fossil energy land, cultivated land and grassland had the greatest impact on ecological sustainability of Guangdong Province, suggesting that the high demand for energy and agricultural products was the main reason for the high ecological footprint.
3. Through analysis and comparison of the cities of Guangzhou, Foshan, Zhuhai, Shaoguan, Meizhou and Qingyuan, it was found that the ecological deficit mainly concentrated in developed areas such as Guangzhou, Foshan and Zhuhai, while Shaoguan, Meizhou, Qingyuan were in ecological surplus for a long time. The ecological footprint was larger than ecological capacity, which might be related to the economic development of Guangdong province. As an export-oriented economy, the introduction of foreign capita, the development of advanced technology and the rapid economic development were shifting the population to the economically developed areas, resulting in a continuous increase in the rate of urbanization. As a result, the ecological footprint of different regions varied greatly.
4. It was estimated that from 2013 to 2018, the per capita trade footprint of Guangdong Province has shown a straight upward trend, which belonged to the overall surplus. Guangdong was a province with complete export of resources and services. Meizhou was a city that fully exports resources and services, while Guangzhou and Foshan were cities that fully receive biological resources services, energy products and services, which was consistent with the positioning of each city. Compared with the per capita ecological footprint before the adjustment, the result was a little bit higher but the trend was consistent. The ecological footprint could be a more realistic and comprehensive reflection of the influence of Guangdong residents to the local ecological system and the sustainable development, as the improved model included products processing and trade between the two cities.
5. This study used the resource yield method to calculate the ecological capacity of Guangdong Province and six prefecture-level cities. Compared with the traditional model, it has overcome the contradiction between spatial exclusivity and yield factor adjustment in the diversification of land use, and more comprehensively considered the characteristics of various types of land, which clearly reflected the local ecological supply of Guangdong Province, and also provided a good basis for trade ecological footprint analysis, making it more accurate to analyze the sustainable development of Guangdong Province; on the other hand, the domestic trade was included in the ecological footprint calculation, and the indirect ecological products and energy trade footprints generated by its trade was also incorporated into the scope of trade adjustments, making its urban-scale ecological footprint more closely match the actual situation, and further ensuring the accuracy of its sustainable development analysis, providing new clues for future regional development analysis.
